# Supplementary material for: Protocol for evaluating the cost-effectiveness of Mongolia’s sugar-sweetened beverages tax using double machine learning
Source: PLoS One. 2025 Jun 10;20(6):e0324378. doi: 10.1371/journal.pone.0324378 (PMC12151437; doi:10.1371/journal.pone.0324378)
Supplement: S1 Table — (DOCX) [file pone.0324378.s001.docx]

**Supporting Information**

**Table S1.** CHEERS checklist

| **Section/topic** | **Item No** | **Reporting Item** | | **Page Number** | |
| --- | --- | --- | --- | --- | --- |
| **Title** | | |  | |  |
| Title | 1 | Identify the study as a cost-effectiveness analysis and specify the intervention (SSB tax in Mongolia). | | 1 | |
| **Abstract** | | |  | |  |
| Abstract | 2 | Provide a structured summary that includes objectives, perspectives, methods, expected outcomes, and conclusions. | | 1 | |
| **Introduction** | | |  | |  |
| Background and objectives | 3 | Provide background and context, study aims, and relevance to Mongolian health policy. | | 1, 2 | |
| **Methods** | | |  | |  |
| Health economic analysis plan | 4 | We will follow the CHEERS and PALISADE checklists during the implementation of the full study. | | N/A | |
| Study population | 5 | Mongolian population aged 15+ in 2023, stratified by age and BMI. | | 7 | |
| Setting and location | 6 | Mongolia; National health system and current NCD and SSB context are described. | | 2 | |
| Comparators | 7 | SSB tax (intervention) vs. no tax (status quo). | | 7 | |
| Perspective | 8 | Societal perspective; includes both health system and out-of-pocket costs. | | 5, 8 | |
| Time horizon | 9 | We evaluate 10-year, 20-year, and lifetime horizons. | | 7 | |
| Discount rate | 10 | 5% annually for both costs and outcomes. | | 8 | |
| Selection of outcomes | 11 | Health outcomes: NCD incidence, QALYs gained, DALYs averted, deaths prevented. | | 7 | |
| Measurement of outcomes | 12 | Modelled from epidemiological transitions and disease risks based on STEPS data. | | 4, 5 | |
| Valuation of outcomes | 13 | Utility (QALY) and disability (DALY) weights sourced from IHME estimates. | | 6 | |
| Measurement and valuation of resources and costs | 14 | Cost data from the 2019 STEPS survey and government health insurance data adjusted to 2023. | | 4, 5, 8 | |
| Currency, price date, and conversion | 15 | All costs will be adjusted to 2023 Mongolian Tugrik and converted to USD using the 2023 exchange rate. | | 8 | |
| Rationale and description of model | 16 | Closed cohort Markov model developed in TreeAge Pro; simulates Healthy–NCD–Death transitions. | | 2, 3, 4 | |
| Analytics and assumptions | 17 | Half-cycle correction applied in the Markov model; price elasticity estimates used to model changes in SSB consumption; cost-effectiveness calculated based on projected changes in weight, BMI, disease incidence, and healthcare costs. | | 3, 5, 6 | |
| Characterizing heterogeneity | 18 | Subgroup analyses were not conducted in the protocol stage; however, future work may stratify by age group and BMI category to assess distributional effects. | | N/A | |
| Characterizing distributional effects | 19 | Not explicitly modelled, but implications discussed in the Discussion. | | N/A | |
| Characterizing uncertainty | 20 | Sensitivity analyses on discount rates, tax pass-through, and reformulation effects. | | 7, 9 | |
| Approach to engagement with patients and others affected by the study | 21 | Not applicable – no direct stakeholder engagement reported. | | N/A | |
| **Results** | 22 – 25 | Not applicable – this is a protocol; model parameters, outcomes, and sensitivity results will be presented in the full study. | | Not yet reported | |
| **Discussion** | 26 | To be included in the final paper. Limitations will address data gaps and modelling assumptions. | | Planned | |
| **Other relevant information** | | | | |  |
| Source of funding | 27 | Funded by Macquarie University. No influence on study design or results. | | 10 | |
| Conflicts of interest | 28 | The authors declare no conflicts of interest. | | 10 | |

Note: This checklist is completed at the protocol stage. Final results and updates will be reported in the full study manuscript, following CHEERS 2022 standards
