# Supplementary material for: Protocol for evaluating the cost-effectiveness of Mongolia’s sugar-sweetened beverages tax using double machine learning
Source: PLoS One. 2025 Jun 10;20(6):e0324378. doi: 10.1371/journal.pone.0324378 (PMC12151437; doi:10.1371/journal.pone.0324378)
Supplement: S2 Table — (DOCX) [file pone.0324378.s002.docx]

**Supporting Information**

**Table S2. PALISADE Checklist**

| **Element** | **Definition** | **Response** |
| --- | --- | --- |
| **Purpose** | Is the purpose of the algorithm clearly stated? Is the implementation in a healthcare setting fair and ethical? | The study will apply machine learning, specifically Double Machine Learning (DML), to estimate the own-price elasticity of sugar-sweetened beverage (SSB) consumption. These elasticity estimates will inform changes in SSB intake due to taxation, which will then be used to project downstream impacts on body weight, BMI, and disease incidence within a Markov model for cost-effectiveness analysis. Implementation will align with ethical principles by enhancing transparency and fairness in public health policymaking. |
| **Appropriateness** | Is there a clear justification that the algorithm is acceptable in the applied context? | DML is appropriate for this context as it enables robust causal inference from observational data by adjusting for confounders. In the absence of randomized pricing interventions in Mongolia, DML provides reliable estimates of price responsiveness, which are critical inputs for modelling the impact of an SSB tax on health and economic outcomes. |
| **Limitations** | Have the strengths and limitations of the algorithm and data been identified? | Key limitations include possible overfitting, reliance on model assumptions, and small sample sizes in local datasets. These will be mitigated through cross-validation, sensitivity analysis, and triangulation of multiple data sources (see Data Characteristics). |
| **Implementation** | Are access, implementation, and resource issues in healthcare settings considered? | DML estimation will be conducted in Stata, with Markov simulations in TreeAge Pro. The workflow is feasible for analysts with training in econometrics and health economics. Results aim to support policy within the resource constraints of the Mongolian healthcare system. |
| **Sensitivity and Specificity** | Has model performance and accuracy been evaluated? | Predictive accuracy will be evaluated via cross-validation within the DML framework. Diagnostic measures (e.g., R-squared for outcome models) and model comparison metrics will guide algorithm selection and validation. Robustness will be tested by varying model specifications and assumptions. |
| **Algorithm Characteristics** | Has the ML mechanism been clearly described, and is the model reproducible? | The DML estimation strategy will be fully documented, including treatment and outcome model specifications, cross-fitting, and inference approach. Reproducibility will be ensured through detailed code, version control, and methodological transparency. |
| **Data Characteristics** | Is the selection of datasets justified, and are their key characteristics documented? | The study will draw on nationally representative sources: 2024 National Nutrition Survey (SSB consumption), 2019 WHO STEPwise Approach to NCD Surveillance (risk and costs), 2023 population statistics, and 2021 Global Burden of Disease data. Data preprocessing, imputation, and documentation will be completed before the final analysis. |
| **Explainability** | Are algorithm outputs interpretable by stakeholders? | Key outputs—such as age-specific transition probabilities and incremental cost-effectiveness ratios (ICERs)—will be presented using clear, non-technical language and accompanied by visuals, including charts and flow diagrams. Key policy messages will be highlighted to ensure accessibility to policymakers, health practitioners, and the general public. |

Note: This checklist has been completed at the protocol stage. The full study manuscript will report Results and methodological updates, adhering to the PALISADE (Predictive Analytics and Learning Algorithms in Health Economics and Outcomes Research) reporting framework. All tools, datasets, and references mentioned herein are cited in the main text and included in the manuscript’s reference list
