# Supplementary material for: Protocol for evaluating the cost-effectiveness of Mongolia’s sugar-sweetened beverages tax using double machine learning
Source: PLoS One. 2025 Jun 10;20(6):e0324378. doi: 10.1371/journal.pone.0324378 (PMC12151437; doi:10.1371/journal.pone.0324378)
Supplement: Attachment — Submitted filename: Response to Reviewers.docx [file pone.0324378.s003.docx]

**Response to Reviewers**

**Reviewer #1**

I have reviewed the manuscript and am pleased with the detailed explanation and its quality. I hope the study went well and look forward to reading the final paper.

**Response:**
Thank you for your positive feedback and support. We appreciate your time in reviewing our work.

**Reviewer #2**

Table 1. Responses to the comments of Reviewer 2

| **Title** | **Comment** | **Response** |
| --- | --- | --- |
| Abstract | Authors say “Given the limited research on the causal relationship between SSB consumption and health outcomes is scarce, we conduct a Cost-Effectiveness Analysis (CEA) using a Markov cohort model and Double Machine Learning (DML) to control for confounding factors and refine causal estimates”. Nowadays a lot of evidence exist about the causal effect of SSB on obesity-related diseases. | We acknowledge that substantial evidence exists on the causal relationship between SSB consumption and obesity-related diseases. To clarify, our study does not seek to establish this relationship but rather to estimate the price elasticity of demand for SSBs using Double Machine Learning (DML) and integrate these estimates into a Markov cohort model for a cost-effectiveness analysis (CEA) of the SSB tax. We have revised the manuscript to reflect this focus more accurately. |
| Data inputs and model specification | To strengthen the study, it would be beneficial to include a figure illustrating the overall modeling process. For example, the change in kcal will be modeled using own price elasticities. For change in obesity,… so the reader is engaged and interested in the methodology. | Thank you for the suggestion. We have added a figure illustrating the logical pathway for modelling the effects of an SSB tax, detailing the sequential process from tax implementation to health outcomes. |
| Data inputs and model specification | 2. Model Specification and Justification: The protocol should provide a more comprehensive description of the Markov cohort model. Some information should be detailed as follows: o “Disease” in the Markov model is obesity or obesity-related diseases? o Describe the state transition probabilities, and assumptions regarding disease progression, mortality and remission. It would be good to include these parameters in table 1. o Mortality rates come from GBD data source, that will conclude in February 2024, but this date already passed. | We have clarified that the model tracks obesity and obesity-related diseases and provided details on state transition probabilities, disease progression, and mortality. Table 1 has been updated to include these parameters for clarity. The mortality rates, previously sourced from 2019 GBD, have been updated to 2023 death statistics from HDC (Health Development Centre) Database Mongolia. Additionally, we have explicitly stated in the manuscript that NCDs are considered irreversible, meaning individuals remain in the disease state once they transition into it. |
| Data inputs and model specification | 3. Is there information about hospitalization of people with obesity? | STEPS 2019 provides self-reported hospitalization data for NCDs, but estimating separate transition probabilities for hospitalization and death would require strong assumptions due to data limitations. Instead, we model individuals with NCDs as a single group while incorporating cost differences for hospitalized and non-hospitalized cases. |
| Data inputs and model specification | 4. Trends in obesity prevalence will be considered, or it will be constant over time? | The model incorporates trends in obesity prevalence, assessing whether they remain stable or decline post-taxation. Sensitivity analyses will evaluate different scenarios, ensuring robustness in cost-effectiveness estimates. |
| Data inputs and model specification | 5. It should clarify how DML is integrated within the modeling framework and how DML will control for confounding? | We have clarified how DML is integrated into the Markov model, using machine learning to estimate the causal effect of the SSB tax on consumption and weight change while controlling for confounders like income, education, and dietary behaviors. These estimates refine transition probabilities, improving the accuracy of the model. |
| Data inputs and model specification | 6. It would be worthwhile to write a paragraph describing Stata command: the ‘ddml’ command.” | We added a paragraph on Stata’s ddml command, explaining its two-stage approach: (1) predicting control variables using LASSO and random forests to reduce model misspecification, and (2) estimating the tax’s causal effect while accounting for confounding. This ensures robust price elasticity estimates, which inform changes in SSB consumption and weight in the Markov framework. |
| Data inputs and model specification | 7. I went to ref 23 and I could not find information on disease costs. Please add this information in an appendix. | We have clarified that out-of-pocket costs related to NCDs are obtained from STEPS 2019, while government health insurance costs are sourced from GAHI. The references have been updated accordingly, and Table 1 now reflects these revised data sources. |
| Data inputs and model specification | 8. Proportion of SSB intake by population: 44.5% I don´t understand this input. Nearly 50% of total energy intake comes from SSBs? Sounds too much. | We acknowledge the need for updated estimates based on the 2024 National Nutrition Survey (NNS), which reports on 2023 consumption. The previously reported 44.5%, based on 2017 National Nutrition Survey (NNS), represented the proportion of adults consuming SSBs daily, not total energy intake from SSBs. However, as we will be using the most recent NNS dataset for consumption estimates, we have excluded this value from the table and will update the analysis accordingly. |
| Data inputs and model specification | 9. Target population: 0-100 years. In other sections it says 2-100. | We have clarified that the target population includes individuals aged 15 years and older, aligning with the 2019 STEPS survey, which provides key health and disease data for this age group. Since our model relies on STEPS data for transition probabilities and healthcare costs, including younger individuals would require additional assumptions. As the focus is on noncommunicable diseases (NCDs), which primarily affect adults, this age threshold ensures data consistency and model accuracy. The Population section has been updated accordingly. |
| Data inputs and model specification | 10. The cost of implementing SSB tax in Mongolia is from an US reference as a % of the SSB tax revenue. While it was done previously, a discussion about this assumption should be presented. | We have revised the manuscript to clarify the assumption regarding SSB tax implementation costs. While official tax policy documents indicate no additional costs for tax collection and implementation, we adopt a tax collection cost of approximately 1% of revenue, along with industry compliance costs, based on prior literature. We have also added a discussion on this assumption and included sensitivity analyses to assess variations in implementation costs and their impact on cost-effectiveness estimates. |
| Data inputs and model specification | 11. An analysis through performance indices would be interesting. With it, you could clarify how a model such as the Random Forest can capture model nonlinearities compared to LASSO. | Thank you for the suggestion. We have clarified how Random Forest and LASSO are used within the DML framework to estimate price elasticity and capture nonlinear relationships in our model. We now compare the ability of Random Forest to capture nonlinearities versus LASSO's regularisation approach, and we have revised the text to highlight the role of these methods in reducing model misspecification. |
| Data inputs and model specification | 12. It would be worth considering techniques such as SHAP (Shapley Additive Explanations), as they facilitate the interpretation of the model results, summarizing the impact of each characteristic on the prediction and how each variable contributes to the predictions. | Thank you for the suggestion. We will apply SHAP to the Random Forest model to enhance interpretability by summarizing the impact of key predictors on price elasticity estimates and consumption changes. We have updated the manuscript to reflect this consideration. |
| Assessment of cost | I could not assess how healthcare expenditures will be estimated. | We have revised the manuscript to clearly describe the estimation of healthcare expenditures using STEPS 2019 for out-of-pocket costs and GAHI for government health insurance costs. Details are now provided in Table 1. |
| Assessment of benefit | I could not assess how DALYs and QALYs will be included in the model. | We have clarified how DALYs and QALYs are incorporated into the model. The manuscript now specifies the utility and disability weights used for NCD cases and how these are applied in the Markov model to estimate the long-term impact of SSB consumption reduction on health outcomes |
| Minor changes | It would be worthwhile adding the definition of the acronym LASSO (Least Absolute Shrinkage and Selection Operator) before mentioning the acronym. | We have added the full definition of LASSO before its first mention. |
